# Equine sarcoids: A clinicopathologic study of 49 cases, with mitotic count and clinical type predictive of recurrence

**DOI:** 10.1177/03009858231209408

**Published:** 2023-11-08

**Authors:** Wilson Karalus, Supatsak Subharat, Geoff Orbell, Bernie Vaatstra, John S. Munday

**Affiliations:** 1 Massey University, Palmerston North, New Zealand; 2 Gribbles Veterinary, Palmerston North, New Zealand

**Keywords:** bovine papillomavirus, clinical type, horse, mitotic count, neoplasia, New Zealand, prognosis, recurrence, sarcoid

## Abstract

Sarcoids are common mesenchymal neoplasms of horses. Although there are few studies in which sarcoids have been followed over a long period of time, sarcoids are considered locally invasive and have been reported to frequently recur following surgical excision. Currently, no histological features have been identified to predict which sarcoids will recur after excision. The present study comprised 49 sarcoids for which histology sections were available and in which the recurrence status of the case was known. Each sarcoid was excised from a different horse. Overall, 12 of the 49 (24%) sarcoids recurred after surgical excision. Mitotic count (MC), cellularity, necrosis, nuclear pleomorphism, and inflammation of the sarcoids were evaluated histologically. Of these, MC correlated with recurrence. Four of 5 (80%) sarcoids with an MC ≥ 20 in 2.37 mm^2^ recurred, which was a significantly higher recurrence rate than that of sarcoids with an MC < 20, 8 of 44 cases recurred (18%), *P* = .0051. Clinical type was also found to correlate with recurrence. Three of 4 (75%) fibroblastic types recurred, which was a significantly higher recurrence rate than that of sarcoids with other clinical types, 9 of 45 cases (18%), *P* < .001. In addition, univariate Cox regression analysis confirmed fibroblastic type and MC ≥ 20 as significant predictors for recurrence (*P* = .016 and *P* = .005, respectively). To the authors’ knowledge, this is the first large study examining recurrence rates in sarcoids, and the first time that histological features have been correlated with recurrence.

Sarcoids are common mesenchymal neoplasms of horses that comprise 12%–67% of all neoplasms in this species.^16,20^ There are no reported sex or age predilections; however, some breeds appear to be predisposed to sarcoid development.^2,7,25^ Sarcoids can develop in any location, but the most common locations include the head and neck, lips, eyelids, ear, extremities, and ventrum.^4,13,38^ Sarcoids are thought to be caused by cross-species infection by bovine papillomaviruses (BPVs), although some aspects of the pathogenesis remain poorly understood.^1,18,25,26,31^

Sarcoids are commonly described as nonmetastatic, locally invasive tumors that often recur.^7,8^ Part of their invasiveness has been attributed to expression of matrix metalloproteinases (MMPs), particularly MMP 1 and MMP 9.^37^ Studies have also identified activation of the platelet-derived growth factor β receptor (PDGFβ-R) and its downstream effects on the p38 mitogen-activated protein kinase (MAPK) pathway, which contributes to fibroblast proliferation and invasion.^36^ In contrast, the expression of pRB, cyclin D1,/ and p53 does not correlate with recurrence rates in sarcoids.^34^ While sarcoids are generally considered tumors that frequently recur after surgical excision, rates of recurrence vary widely within the literature, ranging from 10% to 80%. This may be because many different methods of treatment have been used; however, the high variability of recurrence rates may also be due to the small number of studies that have followed affected horses for a long period of time.^10,14^ One aim of the present study was to determine the frequency with which sarcoids recur after surgical excision.

There are currently few prognostic indicators established for sarcoids. Sarcoids have been subdivided into different clinical types, including occult, verrucose, nodular, fibroblastic, mixed, and malignant/malevolent, and different types have been suggested to have different prognoses.^13^ Occult sarcoids are described as roughly circular alopecic areas that may develop small cutaneous nodules and take on a hyperkeratotic appearance. Verrucose sarcoids have a rougher, hyperkeratotic appearance, with prominent scaling over limited or wider areas. Nodular sarcoids appear as well-defined subcutaneous, spherical nodules, which vary in size and number. These are further subdivided into type A, with no involvement of the skin, and type B, which involve the overlying skin.^10,13^ Fibroblastic types appear as fleshy, raised, ulcerated masses, and have also been further subdivided into type 1, which appear more pedunculated and type 2, which have a broad, locally invasive base.^13^ The mixed category includes sarcoids that have features of 2 or more of the above types in the same tumor.^10^ Although the malignant/malevolent type is described as having lymphatic infiltration and appearing as nodules and cords of palpable tumor, there are few reports of this type in the literature.^10,13,15^ There are currently little data from clinical studies to support the observation that different clinical types have different prognoses, and thus, another aim of the present study was to determine whether different clinical types are more or less likely to recur. Finally, a variety of histological features of sarcoids were examined, and analyzed statistically to identify any correlations between these features and the likelihood of recurrence.

## Material and Methods

### Case Selection and Recruitment of Study Material

Cases that had been histologically diagnosed between 2015 and 2021 as an equine sarcoid were identified by searching the databases of the Pathology Department at Massey University and Gribbles Veterinary Ltd, New Zealand. Search terms used included “equine sarcoid,” “skin neoplasia,” and “skin mass.” Surveys were distributed to submitting clinics to determine patient signalment (age, gender, and breed), location of the sarcoid, the clinical “type” based on the gross appearance of the sarcoid,^13^ treatments used, and the date of any observed recurrence. Each sarcoid came from a different horse and multiple sarcoids from the same horse were not included in the study. If a horse had multiple sarcoids, 1 tumor was randomly selected for inclusion in the study. All cases had to have been surgically removed at least 6 months prior to the end of the clinical data collection period (December 2022). Two horses were excluded as the horse was euthanized or died for unrelated reasons within 3 months of removal of the sarcoid. Other exclusion criteria included the inability to access the tissue block or the tissue block containing insufficient material for evaluation.

### Sarcoid Diagnosis

A diagnosis of equine sarcoid was confirmed in all cases by examination of a hematoxylin and eosin-stained section of the lesion by a veterinary pathologist (JSM) board certified by the American College of Veterinary Pathologists.^24^ This was coupled with the presence of BPV DNA detected via polymerase chain reaction (PCR) testing of the paraffin-embedded blocks, which was performed in a previous study.^24^ BPV DNA was detected in all cases, with most (88%) containing BPV2 DNA, while the remainder either contained BPV2 and BPV1 (2%) or just BPV1 (10%) DNA.^24^ The minimum histological criterion to confirm the diagnosis was an increased density of dermal fibroblasts, which may be arranged in whorls, streams, and/or bundles, any combinations of these patterns, or less commonly other patterns. Other histological criteria assessed to further confirm the diagnosis, included rete peg formation, “picket fencing” (the presence of fibroblasts aligned perpendicular to the epidermal basement membrane), epidermal hyperplasia and hyperkeratosis, surface ulceration, and loss of adnexal structures.^17,30^

### Evaluation of Histological Features

The following histological criteria were assessed and correlated, individually and in combinations, with recurrence outcome: mitotic count (MC), cellularity, necrosis, nuclear pleomorphism, and inflammation. Details of each parameter and the scores assigned are outlined in Table 1. The number of sections trimmed for each case varied depending on the size of the submitted sample; however, only 1 slide was examined from each case as not all cases contained multiple slides to examine. The slide with the most tissue was selected for examination. To evaluate repeatability of each parameter evaluation, after assessment of the histologic criteria by the first pathologist (WK), they were assessed by another pathologist (JSM), who was blinded to the scores of the first pathologist and was instructed to follow the study materials and methods for histologic examination (see below). No knowledge of the recurrence status was known by either pathologist when the histological features were assessed.

**Table 1.** Summary of the histological features and their scoring criteria used to examine the 49 sarcoids in the present study.

Histological Feature	Scoring Criteria
Mitotic count score	No. of mitoses per 2.37 mm^2^
1	≤9
2	10–19
3	≥20
Cellularity score	
1	Loosely packed neoplastic cells overall
2	Tightly packed neoplastic cells overall
Tumor necrosis score	
0	No necrosis
1	≤ 50% necrosis
2	> 50% necrosis
Nuclear pleomorphism score	
1	Mild
2	Moderate
3	Marked
Inflammation score	
0	None
1	Surface inflammation only
2	Surface inflammation and deep inflammation

**Figure 1.**
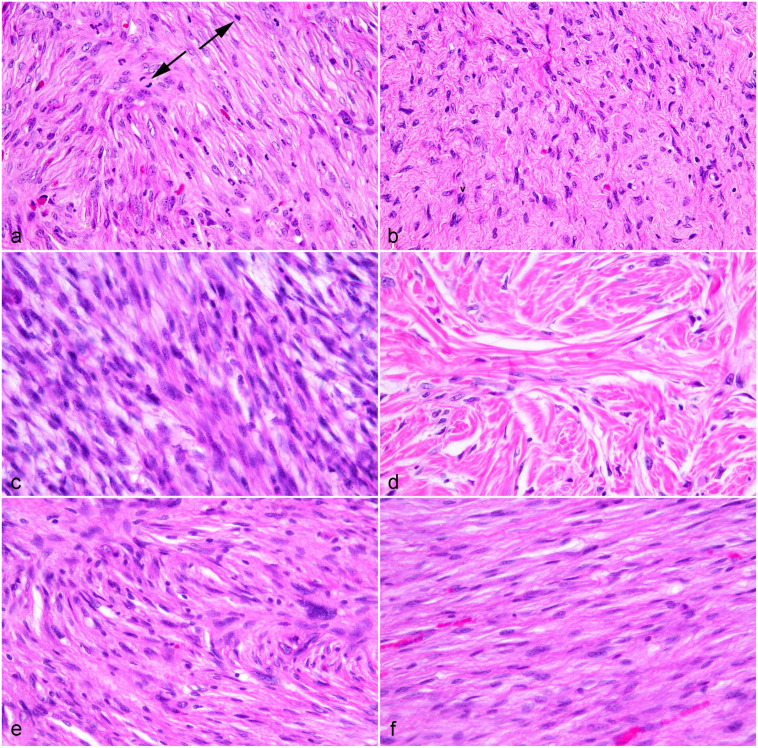
Equine sarcoid. Hematoxylin and eosin (HE). (a) A sarcoid with multiple mitotic figures (arrows). (b) A sarcoid with no identifiable mitotic figures in this field. (c) A highly cellular sample with neoplastic fibroblasts tightly packed and arranged in streams. (d) A sarcoid exhibiting low cellularity with neoplastic fibroblasts loosely packed and arranged around dermal collagen bundles. (e) A sarcoid displaying criteria for marked pleomorphism with wide variation in nuclear size (anisokaryosis), multilobulation, indented nuclei, and karyomegaly. (f) An example of mild pleomorphism for comparison with (e). A sarcoid with mild anisokaryosis and primarily elongate nuclei.

MC was defined as the total number of mitoses counted in 2.37 mm^2^ (10 contiguous, no overlapping 40× objective high-powered fields, 10× ocular FN 22 mm, field of view diameter 0.55 mm at specimen level).^22,23^ Areas of high cellularity with minimal necrosis, inflammation, hemorrhage, or edema were selected for MC assessment. As surface inflammation and ulceration were often present, and can affect the MC, the superficial aspect of the sample was not used to determine MC for all cases.^23^ Sarcoids were subdivided into those with an MC ≤ 9 (score 1), 10–19 (score 2), and ≥ 20 (score 3) (Fig. 1). Cellularity was assessed as primarily loosely packed (score 1) or primarily tightly packed neoplastic cells (score 2) (Fig. 1).

Necrosis was assessed histologically only, as no gross specimens were available for examination in this study. The percentage of necrosis was estimated based on examination of 1 slide per tumor. Sections of each sarcoid that were trimmed and examined were selected by the technician in charge of trimming at the time of processing, with no knowledge of whether the technician was trained to avoid areas of necrosis when trimming samples. The amount of necrosis was evaluated as zero (score 0), ≤ 50% of the tumor (score 1), or > 50% of the tumor (score 2).

Nuclear pleomorphism was assessed based on anisokaryosis, nuclear shape, chromatin pattern, nucleolar size, and multinucleated cells. Cells were examined in a randomly selected 2.37 mm^2^ area. The nuclear features were assessed, and a score was assigned based on the overall appearance. If cells had more features of a higher grade, over a lower grade, the score was categorized as the higher severity. No attempt was made to measure numbers or percentages of cells with the following nuclear features. Definitions of these parameters are provided for each category below and exemplified in Fig. 1. Mild (score 1) pleomorphism was characterized by elongate nuclei with minimal variation in size, hyperchromatic or stippled chromatin, indistinct nucleoli, and absence of multinucleated cells. Moderate (score 2) pleomorphism was characterized by variation in nuclear size with up to 2 times the size of normal dermal fibroblast nuclei, nuclear shape variation, including round to oval shapes, clumped chromatin, distinct nucleoli, infrequent multinucleated cells (1–2 in a randomly selected 2.37 mm^2^ area). Marked (score 3) pleomorphism was characterized by large variation in nuclear size; with a nuclear: cytoplasmic ratio of greater than 1; oval nuclei with 50% or more displaying multilobulation, indentation, angular profiles, or reniform appearance per randomly selected 2.37 mm^2^, clumped chromatin, nucleolar size ≥ 50% of the nucleus size, or ≥ 50% of the cells displaying multiple nucleoli; frequent multinucleated cells (≥ 3 in randomly selected 2.37 mm^2^).

Inflammation was evaluated as 0 (score 0), surface inflammation within the superficial aspect of the tumor only (score 1), or surface/superficial and deep inflammation within the tumor (score 2).

Margins were assessed histologically for each case. Histologically, complete excision was defined as an absence of fibroblasts with histologic evidence of atypia abutting tissue margins. Histological margins were not measured in this study. Histological margin assessment was difficult as fibroblasts that represent a reactive response to the tumor can easily be mistaken for neoplastic cells of the sarcoid, and our definition of histologically complete excision may have overestimated cases of incomplete excision; however, in many cases, the tumor clearly abutted the tissue margin.

## Statistical Analysis

Descriptive analyses were performed. Categorical variables are presented as numbers and percentages, whereas continuous variables are presented as the mean with standard deviation or median with interquartile range. Prior to statistical analyses, the following variables were recategorized: age into 4 groups based on median and interquartile range (< 4, 4–7, 8–10, and > 10 years), and breed into 4 groups (warmbloods, thoroughbreds, standardbreds, and others). The associations between sarcoid recurrence outcome and potential explanatory variables (cellularity, clinical type, inflammation, margins, MC, necrosis, and nuclear pleomorphism), including signalment variables (age, gender, and breed), were tested using chi-squared or Fisher’s exact tests, as appropriate depending on sample sizes. The probability of recurrence of each sarcoid as a function of time was estimated by the Kaplan-Meier method. The association between this measure and explanatory variables was assessed using the pairwise log-rank test. These associations were further assessed by univariate Cox regression models. Multivariate Cox regression models were not fitted, because these models are not recommended for use when there are less than 5 events per variable, and in this study, multiple variables had fewer than 5 sarcoid recurrences.^35^ Data analyses were carried out using R version 4.2.1^27^ with packages *survival*^32^ and *survminer*.^12^ The Cox regression models were tested for proportional hazard assumption using the Schoenfeld test.^28^ Outputs are reported as estimated hazard ratios along with their 95% confidence intervals and *P* value. Statistical significance was set at a *P* value < .05 in all cases.

## Results

### Survey Information

A total of 96 sarcoids were identified, which were submitted from 35 different veterinary clinics across the North and South Islands of New Zealand. Of these, a total of 49 cases contained all relevant information for inclusion in the study. The study therefore comprised 49 sarcoids.

Of the 49 horses, 12 (24%) were warmbloods, 11 (22%) thoroughbreds, 7 (14%) standardbreds, 3 (6%) quarter horses, 3 (6%) sport horses, 3 (6%) Clydesdales, 3 (6%) mixed breeds, 2 (4%) Arabians, 2 (4%) ponies, 1 (2%) Appaloosa, 1 (2%) paint, and 1 (2%) unknown breed. Thirty (61%) of the horses were male, 18 (36%) were female, and the gender of 1 horse was unknown. Of the males, 13 (43%) were geldings. The age of the horses at biopsy ranged from 1 to 16 years (median = 7, first quartile = 4 and third quartile = 10). The ages were recategorized into 4 groups, with 10 horses < 4 years old, 18 horses aged 4 to 7 years, 8 horses between 8 and 10 years old, 12 horses older than 10 years, and 1 age was unknown.

The most common location of the sarcoids was the head and neck (27 cases, 55%), followed by the lower limb, distal to the stifle (10 cases, 21%), upper limb, proximal to the stifle (5 cases, 10%), flank (4 cases, 8%), prepuce, groin, and abdomen (2 cases each, 4%), and a single case from the perineum. No thoracic limbs were affected with sarcoids in this study.

Of the 49 sarcoids, 21 (43%) were reported by the clinician to be nodular type, 14 (29%) were mixed, 10 (20%) were verrucous, and 4 (8%) were fibroblastic. No malignant/malevolent sarcoids were reported.

Surgical excision was the only treatment used for 23 of the 49 sarcoids, with 26 sarcoids receiving additional therapy after surgical excision. Treatments used in addition to surgical excision included a topical combination therapeutic containing 5-fluorouracil, thiouracil, heavy metal salts, and steroid (AW-5-Ludes sourced from Equine Medical Solutions, 9 cases), intralesional 5% 5-fluorouracil (3 cases), topical 5-fluorouracil (Efudix ointment) (4 cases), injectable cisplatin (4 cases), injectable carboplatin (1 case), intralesional cryotherapy (1 case), topical turmeric powder (1 case), and topical bloodroot ointment (Xterra cream, 1 case). One case received multiple treatments (combination of topical Efudix, intralesional cryotherapy and topical AW-5-Ludes), and another case received an unknown chemotherapeutic agent.

### Histological Features

Overall, 34 of 49 (70%) sarcoids had an MC of ≤9, 10 of 49 (20%) had an MC of 10–19, and 5 of 49 (10%) had an MC of ≥20. Cellularity of 13 of 49 sarcoids (27%) was loosely packed, and 36 of 49 sarcoids (73%) were tightly packed. Mild nuclear pleomorphism was visible within 20 of 49 sarcoids (41%), with moderate and marked nuclear pleomorphism visible within 20 of 49 (41%) and 9 of 49 (18%) sarcoids, respectively. Forty-two of 49 (86%) sarcoids had no visible necrosis, 6 of 49 (12%) had necrosis comprising ≤50% of the tissue sample, and 1 of 49 (2%) had necrosis comprising >50% of the tissue sample. Eleven of 49 (22%) sarcoids had no inflammation present, 18 of 49 (37%) had surface inflammation, and 20 of 49 (41%) had surface and deep inflammation.

Nine of 49 (19%) cases appeared completely excised, while 40 of 49 (81%) sarcoids had fibroblasts with histological evidence of atypia extending to tissue margins. Of the 40 cases that were considered incompletely excised, 27 (68%) clearly abutted the tissue margins, while 13 (32%) had more equivocal evidence of fibroblasts with histological evidence of atypia extending to the margins. Of the 23 cases that were treated using only surgical excision, 15 (65%) sarcoids appeared incompletely excised, and 8 (35%) appeared completely excised. Of the 26 cases that received additional treatment, 25 (96%) appeared incompletely excised and 1 (4%) appeared completely excised.

When reevaluated by the second pathologist (JSM), similar histologic parameter scores were produced with no major changes to the statistical analysis of the data.

### Sarcoid Recurrence

**Figure 2.**
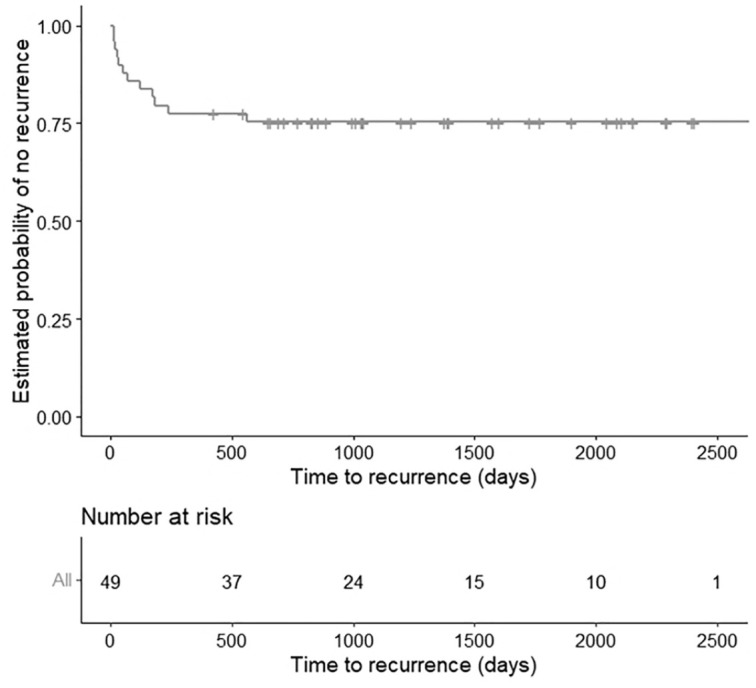
Kaplan-Meier curve for 49 horses with sarcoids illustrating the low overall recurrence observed (12 of 49 [25%]) in the study population.

Overall, 12 of 49 (24%) sarcoids recurred after the tumor was excised (Fig. 2). The recurrence time for the sarcoids ranged between 13 and 561 days. Recurrence was not significantly associated with age, gender, breed, anatomic location, or treatment type (Fisher’s exact test, *P* > .05). However, recurrence was significantly associated with the fibroblastic clinical type (Fisher’s exact test, *P* = .027).

In terms of histological features, recurrence was not significantly associated with cellularity (Fisher’s exact test, *P* = .47), nuclear pleomorphism (Fisher’s exact test, *P* = .19), necrosis (Fisher’s exact test, *P* = .17), or inflammation (Fisher’s exact test, *P* = .41). However, recurrence was significantly associated with MC (Fisher’s exact test, *P* = .020).

One of 9 (11%) sarcoids judged to be completely excised recurred, which was not significantly lower than the 11 of 40 (28%) sarcoids with histologically incomplete excision (*P* = .318) (Table 2). Six of 23 (26%) sarcoids that were treated with surgical excision alone recurred, while 6 of 26 (23%) sarcoids that received any variation of additional treatment(s) recurred. Of the 23 that were treated with surgical excision alone, 5 of 15 (33%) cases that appeared incompletely excised recurred, while 1 of 8 (12.5%) cases that appeared completely excised recurred. Within the 26 sarcoids treated with additional treatment(s), 6 of 25 (24%) sarcoids with histologically incomplete excision recurred, and no recurrence was observed in the single remaining case with histologically complete excision. One of 9 cases treated with AW-5-Ludes recurred, 1 of 4 cases treated with cisplatin recurred, 3 of 4 cases treated with Efudix recurred, and 1 case treated with carboplatin recurred. There was no statistically significant difference in recurrence, regardless of the treatment used (Fisher’s exact test, *P* = .334).

**Table 2.** Univariate Cox proportional hazards regression model reporting the estimated hazard ratios, 95% CI and *P* value.

Variable	Recurrence Events (12 of 49)	Hazard Ratio (95% CI)	*P* value
Cellularity			
Score 1	2 of 13	Ref.	.360
Score 2	10 of 36	2.03 (0.45–9.29)	
Clinical type			
Verrucous	2 of 10	Ref.	
Fibroblast	3 of 4	9.21 (1.51–56.25)	**.016**
Mixed	5 of 14	1.94 (0.38–10.04)	.427
Nodular	2 of 21	0.46 (0.07–3.29)	.442
Inflammation			
Score 0	2 of 11	Ref.	
Score 1	3 of 18	0.84 (0.14–5.03)	.849
Score 2	7 of 20	2.17 (0.45–10.45)	.335
Margins			
Complete	1 of 9	Ref.	
Incomplete	11 of 40	2.84 (0.37–21.98)	.318
Mitotic count			
0–9	6 of 34	Ref.	
10–19	2 of 10	1.19 (0.24–5.91)	.830
>20	4 of 5	6.06 (1.70–21.59)	**.005**
Necrosis			
Score 0	9 of 42	Ref.	
Score 1	2 of 6	1.56 (0.34–7.21)	.571
Score 2^a^	1 of 1	—	—
Nuclear pleomorphism			
Mild	3 of 20	Ref.	
Moderate	8 of 20	3.06 (0.81–11.57)	.099
High	1 of 9	0.76 (0.08–7.34)	.816

Abbreviation: CI, confidence interval.Bold values indicate statistical significance set at a *P* value < .05 in all cases.

a Not included in the analysis as there is only a single observation for this group.

There was no significant difference in the rate of recurrence between sarcoids treated only with surgical excision and sarcoids that were treated by surgical excision followed by additional treatment (Pearson’s chi-squared test, *P* = .807).

### Effects of Pathological Indexes on Sarcoid Recurrence

**Figure 3.**
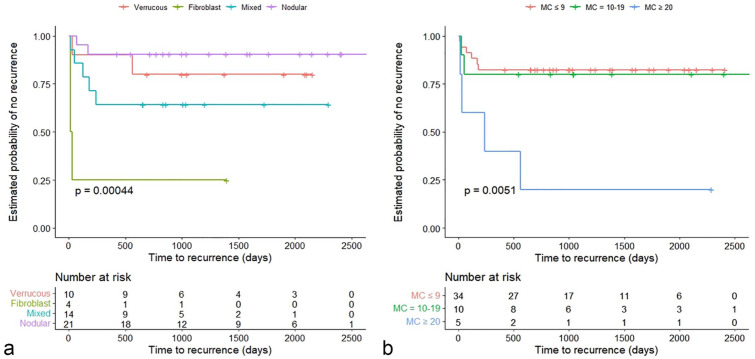
Kaplan-Meier curves for 49 horses with sarcoids. (a) Kaplan-Meier curve for 49 horses with sarcoids examined according to the clinical type. The difference in recurrence of each clinical type is illustrated, with fibroblastic types significantly more likely to recur than verrucous, mixed, or nodular types (log-rank test: *P* = .00044). (b) Kaplan-Meier survival curve for 49 horses with sarcoids illustrating the difference in recurrence among cases with ≤ 9 mitoses/2.37 mm^2^, 10–19/2.37 mm^2^, and ≥ 20/2.37 mm^2^ (log-rank test: *P* = .005). MC, mitotic count.

The probability of recurrence of each sarcoid as a function of time was estimated by the Kaplan-Meier method (Fig. 2). There was no significant effect of cellularity, nuclear pleomorphism, necrosis, or inflammation on the probability of recurrence (log-rank, *P* > .05). From a pairwise comparison with Bonferroni correction, the probability of recurrence was significantly higher for a fibroblastic sarcoid than for a nodular sarcoid (log-rank, *P* < .001) and trends were observed when compared with verrucous types (log-rank, *P* = .117) and mixed types (log-rank, *P* = .165). There was no statistical difference between other clinical types (log-rank, *P* > .05) (Fig. 3a).

In addition, from a pairwise comparison with Bonferroni correction, the probability of recurrence was significantly higher in MC ≥ 20 group than that of MC ≤ 9 group (log-rank, *P* = .005) and suggested a trend when compared with MC 10–19 group (log-rank, *P* = .1), while the difference between MC ≤ 9 and MC 10–19 was not statistically significant (log-rank, *P* = 1.0) (Fig. 3b).

The factors associated with the probability of recurrence of each sarcoid were analyzed by univariate Cox regression models. The estimated hazard ratios, 95% confidence intervals, and *P* value of univariate analyses are reported in Table 2. Clinical type (fibroblastic) and MC (≥ 20) were both significantly associated with the probability of recurrence of a sarcoid in univariate analyses (*P* = .016 and *P* = .005, respectively; Table 2). The Schoenfeld test was performed to test for proportional hazard assumption. For clinical type, the Schoenfeld test yielded a *P* value of .42, suggesting no violation of the proportional hazard assumption. Similarly, for MC, the Schoenfeld test yielded a *P* value of .58, indicating no violation of the proportional hazard assumption.

## Discussion

In this study, sarcoids with an MC ≥ 20 were more likely to recur after excision than sarcoids with an MC ≤ 9. While this potential prognostic indicator needs additional validation in other series of sarcoids, the results suggest assessment of MC, a parameter that is comparatively easily determined by pathologists, could be a useful indicator of recurrence. Identifying sarcoids that are more likely to recur will be useful for clinicians to determine the most appropriate monitoring and treatment following excision of a sarcoid. To the authors’ knowledge, no previous study has evaluated the use of histological features to predict the likelihood of recurrence of an equine sarcoid.

It is often reported in the literature that the clinical type of a sarcoid is an important prognostic indicator, with the fibroblastic and more complex mixed types viewed as more aggressive and more likely to recur.^10,13,15^ However, there are little published data on these claims, and most reports appear anecdotal. The present study aimed to investigate these claims further and found that the clinical type was a strong predictor of recurrence, in particular the fibroblastic type. However, this observation needs further validation on other series of sarcoids as only 4 sarcoids were classified as fibroblastic in the current study. In addition, as the clinical types were assessed by a number of different veterinarians, the repeatability of this classification is unknown. Other factors, including the tumor size, and number of lesions on each horse were not assessed in the present study, and so that, it remains possible these factors may also determine the likelihood of recurrence.^4,15,19^

Only a quarter of sarcoids recurred after surgical excision in this study. Similarly, low rates of recurrence were observed in sarcoids that were classified as incompletely excised and in sarcoids that received no additional therapy after excision. This is in contrast to previous studies that have reported that around 70% of sarcoids recur after surgical excision.^15,21^ A potential cause for this difference in recurrence rates includes the low number of sarcoids that were removed by surgical excision included in previous studies.^15,21^ In addition, some of the previous studies were performed at referral clinics, suggesting the possibility that these horses were referred due to recurrent or more extensive sarcoids. However, consistent with the present study, recurrence rates of 18% were reported in a study of 57 sarcoids that had been surgically excised.^19^ In this previous study, the low rates of sarcoid recurrence were attributed to the careful selection of sarcoids for surgical excision, wide surgical margins, and a “non-touch” surgical technique to avoid auto-inoculation.^19^ While wide margins and special surgical techniques may be beneficial, the results of the present study suggest that even incompletely excised sarcoids may be much less likely to recur than historically reported. Rates of recurrence between 10% and 20% have also been reported in some evaluations of novel treatments, including using diode laser therapy, electrosurgical excision, radiotherapy, and intralesional cisplatin.^6,9,11,29,33^ While additional studies are required, the results of the present study suggest that the benefit of these novel treatments in preventing sarcoid recurrence could be less marked than previously thought.

Just over half of the sarcoids in the current study were treated with additional therapy after surgical excision. However, the rates of recurrence between untreated and treated cases were not statistically different. These results suggest limited benefit from additional treatments. However, after consultation with many of the clinicians involved with these cases, some explanations for deciding to choose additional treatment(s) included large tumor size, difficulty in surgical excision due to the anatomic location, or grossly observed invasiveness. This is not an all-inclusive list, and further studies on the treatment(s) used and their results are warranted. Comparison between the different treatments used in this study was limited due to the small number of horses in each treatment group, and further statistical analysis could not be made. Regardless, the results of the study suggest that even without additional treatment, the majority of sarcoids will not recur after surgical excision.

In the present study, completeness of excision was not significantly associated with recurrence. This could be due to difficulties in assessing surgical margins histologically with cells interpreted as being part of the sarcoid actually representing a fibroblastic response to the tumor. This represents a limitation to the study in that we were unable to clearly delineate between a neoplastic fibroblast and a reactive fibroblast histologically. However, while this could have affected the margin assessment for a proportion of the sarcoids examined, for others, the sarcoids appeared as obvious masses of proliferating cells abutting the surgical margin. This method is also commonly used by pathologists when examining sarcoids, and the distance is not necessarily measured. However, whether the tumor-free distance in millimeters influences recurrence rates could be a focus for future studies. Previous studies have reported between 30% and 50% of sarcoids resolve without treatment.^3,4,19^ It is therefore possible that some of the incompletely excised sarcoids did not recur because the tumor spontaneously resolved prior to the remaining sarcoid cells progressing into a mass that was large enough to be clinically visible.

Other histological criteria assessed were cellularity, necrosis, nuclear pleomorphism, and inflammation. However, none of these features significantly predicted recurrence. These features are subjective and difficult to reproduce accurately and consistently and are a common source of error in studies focusing on prognostic indicators. However, to mitigate this, another pathologist was provided the materials and methods section for training, and blinded to the authors first score and the recurrence status of the cases. All parameters were repeatable and similar scores were produced.

Another limitation of this study is the amount of right censoring experienced when analyzing the data statistically. Right censoring occurs when the observed event (recurrence) does not occur during the course of the study. Thirty-seven (75.5%) of the horses did not have any observable recurrence within the study period of 7 years. In addition, the horses in this study were not all followed over the same time period (some horses entered the study later than others). This is difficult to avoid in retrospective studies on survival or recurrence due to the variance in the times that cases are added to the study population. However, a criterion for inclusion in the study was that each sarcoid had to have had at least 6 months of follow-up after the first surgical treatment. This was chosen as it aligns with other studies in the literature and first recurrence often occurs within the first 3–6 months following surgical excision.^4,5,20^ A prospective study on recurrence in equine sarcoids may produce more accurate results and represents an area of possible future research.

This study is the first to evaluate whether recurrence of an equine sarcoid can be predicted by the histological features of the tumor. In this study, sarcoids classified as fibroblastic were more likely to recur than other clinical types and those with an MC ≥ 20 were more likely to recur than sarcoids with an MC ≤ 9. This study also demonstrated that a relatively small proportion (24%) of sarcoids recurred after surgical excision when compared with previous studies.^4,15,19^ In addition, neither the completeness of histologically assessed surgical excision nor the use of additional treatments significantly altered recurrence rates.
